# Electrical Stimulation of the Lateral Entorhinal Cortex Causes a Frequency-Specific BOLD Response Pattern in the Rat Brain

**DOI:** 10.3389/fnins.2019.00539

**Published:** 2019-05-24

**Authors:** Karla Krautwald, Liv Mahnke, Frank Angenstein

**Affiliations:** ^1^Functional Neuroimaging Group, Deutsches Zentrum für Neurodegenerative Erkrankungen (DZNE), Magdeburg, Germany; ^2^Department Functional Architecture of Memory, Leibniz Institute for Neurobiology (LIN), Magdeburg, Germany; ^3^Leibniz Institute for Neurobiology (LIN), Magdeburg, Germany; ^4^Medical Faculty, Otto von Guericke University Magdeburg, Magdeburg, Germany

**Keywords:** BOLD fMRI, *in vivo* electrophysiology, limbic system, prefrontal cortex, amygdala, piriform cortex

## Abstract

Although deep brain stimulation of the entorhinal cortex has recently shown promise in the treatment of early forms of cognitive decline, the underlying neurophysiological processes remain elusive. Therefore, the lateral entorhinal cortex (LEC) was stimulated with trains of continuous 5 Hz and 20 Hz pulses or with bursts of 100 Hz pulses to visualize activated neuronal networks, i.e., neuronal responses in the dentate gyrus and BOLD responses in the entire brain were simultaneously recorded. Electrical stimulation of the LEC caused a wide spread pattern of BOLD responses. Dependent on the stimulation frequency, BOLD responses were only triggered in the amygdala, infralimbic, prelimbic, and dorsal peduncular cortex (5 Hz), or in the nucleus accumbens, piriform cortex, dorsal medial prefrontal cortex, hippocampus (20 Hz), and contralateral entorhinal cortex (100 Hz). In general, LEC stimulation caused stronger BOLD responses in frontal cortex regions than in the hippocampus. Identical stimulation of the perforant pathway, a fiber bundle projecting from the entorhinal cortex to the dentate gyrus, hippocampus proper, and subiculum, mainly elicited significant BOLD responses in the hippocampus but rarely in frontal cortex regions. Consequently, BOLD responses in frontal cortex regions are mediated by direct projections from the LEC rather than via signal propagation through the hippocampus. Thus, the beneficial effects of deep brain stimulation of the entorhinal cortex on cognitive skills might depend more on an altered prefrontal cortex than hippocampal function.

## Introduction

Deep brain stimulation has become an emerging tool for the treatment of cognitive decline during the progression of neurodegenerative diseases. In particular, stimulation of the fornix and entorhinal cortex has shown promising results, whereas stimulation of the hippocampus had so far presented mixed results (Suthana et al., 2012; Hardenacke et al., 2013; Hescham et al., 2013; Suthana and Fried, 2014; Bick and Eskandar, 2016). In mice, entorhinal cortex stimulation has been shown to increase adult neurogenesis in the dentate gyrus (Stone et al., 2011) and to decrease amyloid plaque load in the hippocampus (Xia et al., 2017); thus, entorhinal cortex stimulation might act by improving hippocampal signal processing. On the other hand, the EC also directly interacts with prefrontal cortex regions during memory consolidation and retrieval (Takehara-Nishiuchi, 2014), so entorhinal cortex stimulation might affect cognitive functions by modifying signal processing in the prefrontal cortex. To reveal the brain-wide neuronal networks that are affected by deep brain stimulation of the entorhinal cortex, stimulation can be performed during fMRI measurements.

In previous studies, we employed electrical perforant pathway stimulation to activate the hippocampal formation, i.e., orthodromic neurons in the dentate gyrus, hippocampus proper (CA1-CA3), subiculum, and antidromic neurons in the entorhinal cortex. It turned out that, under certain stimulation conditions, significant fMRI responses were also triggered in several cortical and subcortical structures outside the hippocampal formation, in particular in the septal area and in the nucleus accumbens, medial prefrontal cortex/anterior cingulate, basolateral amygdala, and VTA/substantia nigra (Helbing et al., 2013; Riemann et al., 2017). Of particular importance is the observed functional interaction between the hippocampal formation and the prefrontal cortex, which is thought to be crucially involved in memory storage and retrieval processes (Euston et al., 2012; Preston and Eichenbaum, 2013; Schlichting and Preston, 2016; Eichenbaum, 2017). Co-activation of the medial prefrontal cortex (mPFC) and other cortical areas critically depends on the applied stimulation frequency. In particular, stimulation with bursts of high-frequency pulses (i.e., at 100 Hz) trigger significant fMRI responses in the mPFC, whereas frequencies lower than 20 Hz are ineffective. Also, the quality of previous stimulation influences the distribution of significant BOLD responses during subsequent stimulation (Angenstein et al., 2013; Krautwald et al., 2013; Helbing et al., 2017). Previous studies have revealed that direct electrical stimulation of the hippocampal CA3 region with 2, 5, or 40 Hz was insufficient to trigger significant fMRI responses in the mPFC (Scherf and Angenstein, 2015, 2017; Moreno et al., 2016), whereas 10–20 Hz was effective (Moreno et al., 2016). However, 10 and 20 Hz stimulation also elicited strong fMRI responses in the entorhinal cortex, whereas no significant fMRI responses in the entorhinal cortex were observed during all other stimulation frequencies. Similarly, direct electrical stimulation of Schaffer collaterals with high-frequency (200 Hz) pulse bursts caused significant positive fMRI responses in the ipsi- and contralateral hippocampus but, if at all, only negative BOLD responses in the mPFC (Bovet-Carmona et al., 2018); again, this stimulation did not trigger significant BOLD responses in the entorhinal cortex. Based on these results, we hypothesize that high-frequency (i.e., in the high gamma frequency range) pulse stimulation of the perforant pathway stimulation elicits significant positive BOLD responses in the prefrontal cortex mainly via entorhinal cortex projections. Thus, high gamma frequency-related activation patterns in the entorhinal cortex are more effectively transferred to the mPFC via direct projections rather than via the hippocampus proper/subiculum. To test this assumption, we stimulated either the right perforant pathway or the right lateral entorhinal cortex (LEC) with 5, 20, and 100 Hz pulse sequences and monitored stimulus-related fMRI signal changes in the entire brain and simultaneously recorded field potentials in the right dentate gyrus. If BOLD responses in the frontal cortex are preferentially controlled by LEC projections, electrical stimulation of the LEC should elicit stronger BOLD responses than similar electrical perforant pathway stimulation.

## Materials and Methods

Animals were cared for and used according to a protocol approved by the animal experiment committee and in conformity with the European convention for the protection of vertebrate animals used for experimental purposes and institutional guidelines 86/609/CEE, November 24, 1986. The experiments were approved by the animal care committee of the State of Saxony-Anhalt (No.: 42502-2-1218 DZNE) and were performed according to the ARRIVE (Animal Research: Reporting *in vivo* Experiments) guidelines. Male Wistar-Han rats were housed individually at a constant temperature (23°C) and maintained on a controlled 12:12 h light/dark cycle with food and tap water available ad libitum. For all experiments, 28 rats (LEC 8, PP 20) were scanned. One rat with an implanted electrode in the LEC had to be removed from measurements because of contact problems.

### Surgery and Electrode Implantation

The experimental approach to combine electrical stimulation with field recordings in the dentate gyrus and BOLD responses in the whole brain was similar to previous studies using only perforant pathway stimulation (Helbing et al., 2017; Riemann et al., 2017). Nine-week-old male Wistar-Han rats (270–330 g) were deeply anesthetized with Nembutal (40 mg/kg, i.p.) and placed in a stereotactic frame. To stimulate the LEC, a bipolar stimulation electrode (114 μm in diameter, Teflon-coated tungsten wire, A-M Systems) was implanted at the following position: AP: −7.0 mm, ML: 5.1 mm from Bregma; DV 6.2 mm. To stimulate the perforant pathway, a bipolar stimulation was implanted with the following parameters: AP: −6.9 mm; ML + 4.1 mm from Bregma; DV 2.3–3.0 mm from the dural surface of the right hemisphere according to the atlas of Paxinos and Watson (Paxinos and Watson, 1988).

To measure the electrophysiological response in the dentate gyrus, a monopolar recording electrode was placed in the granular cell layer (AP: −4 mm, ML: 2.3 mm; DV: 2.9-3.5 mm from dura). The correct placement of stimulation and recording electrodes during the implantation was verified by measuring monosynaptic field potentials. Silver-wire grounding and reference electrodes were placed on the dura of the left skull and fixed with plastic screws and dental cement.

### Electrical Stimulation and Functional MRI (fMRI)

All combined electrophysiology/fMRI measurements were performed on a 4.7 T Bruker Biospec 47/20 animal scanner (free bore of 20 cm) equipped with a BGA 09 (400 mT/m) gradient system (Bruker BioSpin GmbH, Ettlingen, Germany). A 50 mm Litzcage small animal imaging system (DotyScientific Inc., Columbus, SC, United States) was used for RF signal reception.

All animals were initially anesthetized with isoflurane (2.0%; in 50:50 N_2_:O_2_; v:v), fixed into the head holder, and connected to recording and stimulation electrodes. Then isoflurane concentration was reduced to 1.5% and a bolus of medetomidine (Dorbene, Zoetis GmbH, Berlin, Germany, 50 μg/kg) was subcutaneously injected. Ten minutes later, the isoflurane concentration was further reduced to 0.4% and continuous application of medetomidine (100 μg/kg per h s.c.) was started. Five minutes later, isoflurane application was completely switched off.

All necessary MRI and electrophysiological adjustments for the simultaneous fMRI experiment were set in parallel before the measurements began. Breathing, heart rate, and oxygen saturation were monitored throughout the experiment by an MRI-compatible pulse oximeter (MouseOX^TM^; Starr Life Sciences Corp., Pittsburgh, PA, United States). Heating was provided from the ventral site. All measured physiological parameters remained constant during the fMRI session, i.e., electrical LEC or perforant pathway stimulation had no effect on breathing (about 60 breath/min), heart rate, and systemic blood oxygen saturation.

**FIGURE 1:**
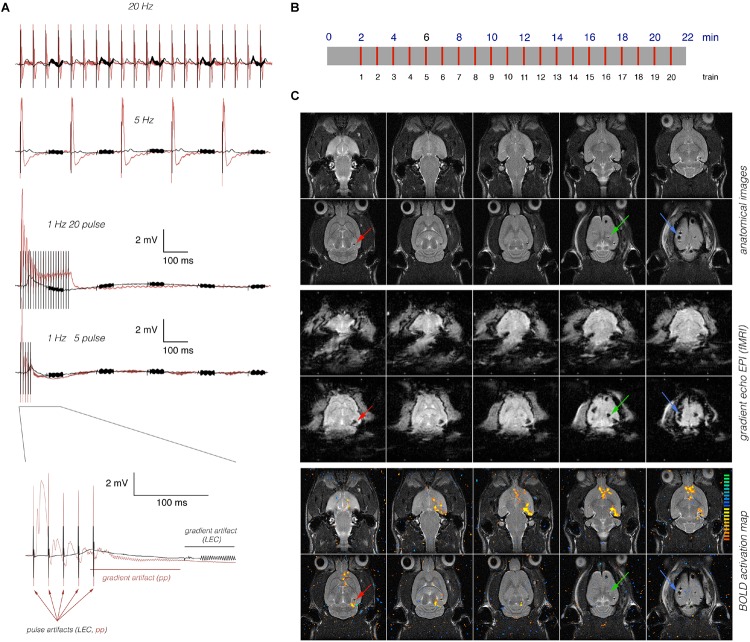
Concurrent measurements of electrophysiological and fMRI data. **(A)** Electrophysiological recordings in the right dentate gyrus during stimulation of the right LEC (black line) or right dentate gyrus (red line). Neuronal responses to LEC stimulation were smaller and delayed when compared to perforant pathway stimulation. Arrows indicate stimulation artifacts (pulses), and the red [perforant pathway (pp) stimulation] or black (LEC stimulation) line indicates the location of the scanner-induced gradient artifacts. **(B)** Scheme of the general stimulation protocol. In all experiments, 20 identical 8 s long stimulation periods (indicated by red lines) were applied. **(C)** Anatomical images (top) reveal the presence of the stimulation electrode (red arrow), the recording electrode (green arrow), and the grounding electrode (blue arrow). The artifacts induced by the electrodes are more pronounced in the corresponding gradient-echo EPI (middle) that are used for fMRI. Overlay of the BOLD activation map on the anatomical image revealed significant BOLD activation in long distance from the stimulation and recording electrode (bottom).

The LEC or perforant pathway was stimulated with 20 consecutive stimulation trains; the first train was presented 2 min after starting the fMRI session. Each train lasted 8 s and was followed by 52 s of rest. At the beginning of every minute, one stimulation train was applied (Figure 1). Bipolar pulses with a duration of 0.2 ms and an intensity of 500 μA were used to stimulate the LEC. This intensity was required to elicit field responses in the dentate gyrus (Figure 1). The intensity of perforant pathway stimulation (between 200 and 300 μA) was determined according to the measured input-output curve as described earlier (Riemann et al., 2017). Trigger pulses that were generated by the scanner at the beginning of every volume, i.e., every 2 s, were used to synchronize fMR image acquisition and electrophysiological stimulation. The total scanning time for one fMRI experiment was 22 min.

The electrophysiological responses during stimulation were recorded with a sample rate of 5000 Hz, filtered between 1 and 5000 Hz by using a differential amplifier EX 4-400 (Science Products, Hofheim, Germany), transformed by an analog-to-digital interface (power-CED, Cambridge Electronic Design, Cambridge, United Kingdom), and stored on a personal computer. During the low sampling rate that was required for the long-lasting recordings during the entire stimulation train, the shape of the recorded population spike was similar to the shape of the initial response that was measured during the input output curve at a sampling rate of 13000 Hz because the underlying signals did not contain frequencies higher than 1 kHz.

No further processing filter was needed because the minor artifacts of the imaging system were small in comparison with the recorded field potential.

For anatomical images, 10 horizontal *T*_2_-weighted spin-echo images were obtained with a *RARE* sequence [rapid acquisition relaxation enhanced (Hennig et al., 1986)] with the following parameters: TR 4000 ms, TE 15 ms, slice thickness 0.8 mm, FOV 37 mm × 37 mm, matrix 256 × 256, RARE factor 8, and number of averages four. The total scanning time was 8 min 32 s. Functional MRI (fMRI) was performed with a gradient-echo EPI (echo planar imaging) sequence with the following parameters: TR 2000 ms, TE 24 ms, slice thickness 0.8 mm, FOV 37 mm × 37 mm, matrix 92 × 92, and a total scanning time per frame of 2 s. The slice geometry (i.e., ten horizontal slices) was identical to the previously obtained anatomical spin-echo images.

### Data Processing and Analysis

The functional data were loaded and converted into BrainVoyager data format. Similar to previous studies (Helbing et al., 2017; Riemann et al., 2017), a standard sequence of preprocessing steps implemented in the BrainVoyager QX 2.6.1 software (Brain Innovation, Maastricht, Netherlands) such as slice time correction, motion correction (trilinear interpolation and reduced data using the first volume as a reference), and temporal filtering (FWHM 3 data points) were applied to each data set. In contrast to these previous studies, no BOLD baseline correction was performed. Because the reconstruction of the fMRI images resulted in a 128 × 128 matrix (instead of the 92 × 92 imaging matrix), spatial smoothing (Gaussian filter of 1.4 voxel) was added.

#### GLM Analysis

Each individual functional data set was used for a multiple-subject GLM analysis implemented in BrainVoyager QX 2.6.1 software. Functional activation was analyzed by using the correlation of the observed BOLD signal intensity changes in each voxel with a predictor (hemodynamic response function), generated from the given stimulus protocol. Based on this, the appropriate 3D activation map could be generated. To calculate the predictor, the square wave representing stimulus on and off conditions was convolved with a double gamma hemodynamic response function (onset 0 s, time to response peak 5 s, time to undershoot peak 15 s). To exclude false-positive voxels, a correction for serial correlation (csc) was performed (implemented in the BrainVoyager QX 2.6.1 software), and we only considered those with a significance level above the threshold set by calculating the false discovery rate (FDR) with a q-value of 0.001 (which corresponds to a t value greater than 4.3 or *p* < 1.6 × 10^−5^). Adjoining significantly activated voxels were summarized as a cluster of activation (cluster size threshold: 30 voxel), and their averaged BOLD time series was visualized. As a GLM analysis is spatially unrestricted, these clusters are not necessarily identical with individual brain structures, so specific brain structures that are part of an individual activation cluster are mentioned for each cluster.

#### VOI Analysis

Each individual functional imaging data set was aligned to a 3D standard rat brain using the 3D volume tool implemented in BrainVoyager QX 2.6.1 software. Individual VOIs were right/left hippocampus, right/left dorsal hippocampus, right/left nucleus accumbens, right/left dorsal striatum, anterior cingulum/prefrontal cortex, septum, right basolateral amygdala, right piriform cortex, and ventral tegmental area/substantia nigra (VTA/SN). These VOIs were marked according to the stereotactic rat brain atlas (Paxinos and Watson, 1988) in the 3D standard rat brain. The averaged BOLD time series of all voxels located in one VOI was then calculated for each individual animal using the volume-of-interest analysis tool implemented in the BrainVoyager QX 2.6.1 software. Each individual BOLD time series was normalized using the averaged BOLD signal intensity of 100%. All normalized BOLD time series were then averaged and depicted as mean BOLD time series ± SEM. Based on the calculated BOLD time series, event-related BOLD responses were calculated by measuring the signal intensities starting six frames before stimulus onset (−12 s until 0 s), during stimulus presentation (between 0 and 8 s, which corresponds to four frames), and the following 15 frames (8–38 s) after the end of the stimulus (Figure 1). To avoid the confounding effect of putative variations in baseline BOLD signal intensities on the calculated BOLD response (i.e., BOLD signal_stimulus_/BOLD signal_baseline_ × 100%), each BOLD response was related to BOLD signal intensities of the stimulus over the preceding 12 s.

## Results

The right LEC was stimulated for 8 s, either with continuous 5 or 20 Hz pulse trains or with one burst of 5 or 20 high-frequency (100 Hz) pulses per s. To compare putative frequency-dependent specific BOLD activation patterns, the same animals were used for all four different stimulation conditions, so differences in the BOLD activation pattern were not caused by different locations of the stimulation electrode. Individual stimulation protocols were performed at intervals of one week and in different orders to avoid stimulation dependent signal habituation. All stimulation conditions triggered measurable electrophysiological responses in the right dentate gyrus. Whereas continuous 5 or 20 Hz pulses triggered individual field responses to all consecutive pulses, high-frequency pulse bursts triggered one contiguous response that was fairly similar when these pulse bursts consisted of 5 or 20 pulses. In contrast, electrical stimulation of the right perforant pathway with these stimulation protocols elicited substantially stronger electrophysiological responses in the dentate gyrus (Figure 1). Thus, perforant pathway stimulation was more efficient to activate neurons in the dentate gyrus. All electrophysiological recordings were simultaneously performed during the fMRI measurements, so the electrophysiological responses reflect neuronal activities in the dentate gyrus during formation of the corresponding BOLD responses.

### Electrical Stimulation of the Lateral Entorhinal Cortex With 5 Pulses per Second

**FIGURE 2:**
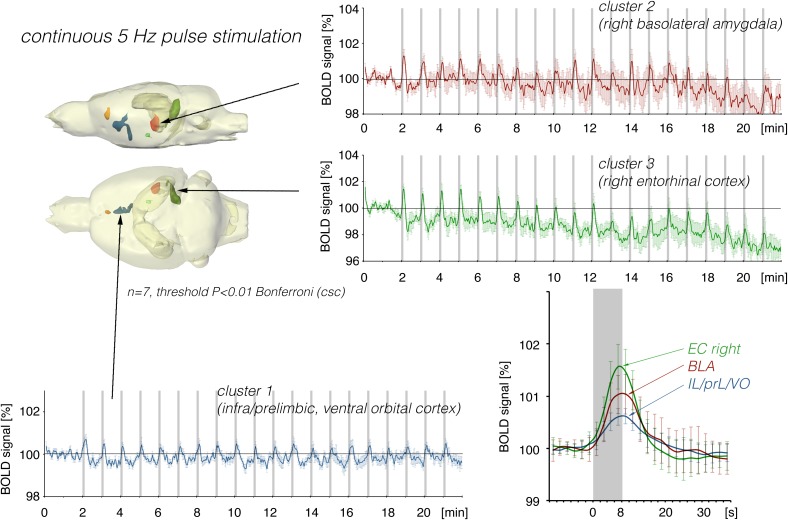
Summary of BOLD responses induced by stimulation of the right LEC with repetitive 5 Hz pulse trains. Spatial distribution of significantly activated voxels revealed the presence of three main clusters of activation. BOLD time series of each individual cluster and event related averaging of all responses to train 3–20 indicate that the strongest BOLD response was induced in the lateral entorhinal cortex, whereas the smallest BOLD response was induced in ventral frontal cortical regions. The gray boxes indicate the time periods of stimulation.

Repetitive electrical stimulation of the right LEC with continuous 5 Hz pulses for 8 s elicited scattered but significant BOLD responses in the right ventral hippocampus, right basolateral amygdala (BLA), and infra-, prelimbic, and dorsal peduncular cortex (IL, PrL, DP, Figure 2). Whereas repetitive stimulation caused a continuous decline in baseline BOLD signals in the right entorhinal cortex, baseline BOLD signals remained fairly stable in all other regions.

**FIGURE 3:**
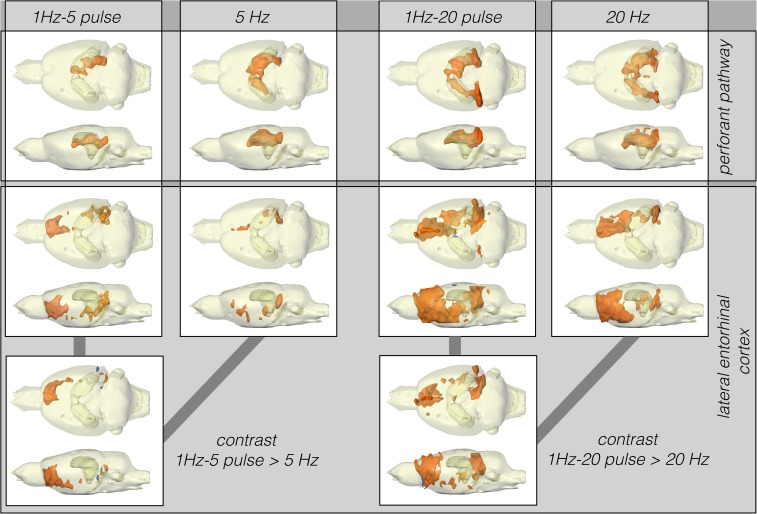
Summary of BOLD activation pattern induced by stimulation of the right perforant pathway (top) and LEC (bottom) with different stimulation protocols. Significant differences in BOLD activation between continuous (5 or 20 Hz pulses) and high-frequency pulse burst (5 or 20 pulses) stimulation of the right LEC (bottom). The same number of pulses applied as bursts with an inter-pulse interval of 10 ms (100 Hz) triggers an expanded BOLD response in frontal regions.

In contrast to LEC stimulation, stimulation of the perforant pathway with 5 Hz elicited clear BOLD responses in the right and left dorsal hippocampus and right entorhinal cortex (Figure 3).

**FIGURE 4:**
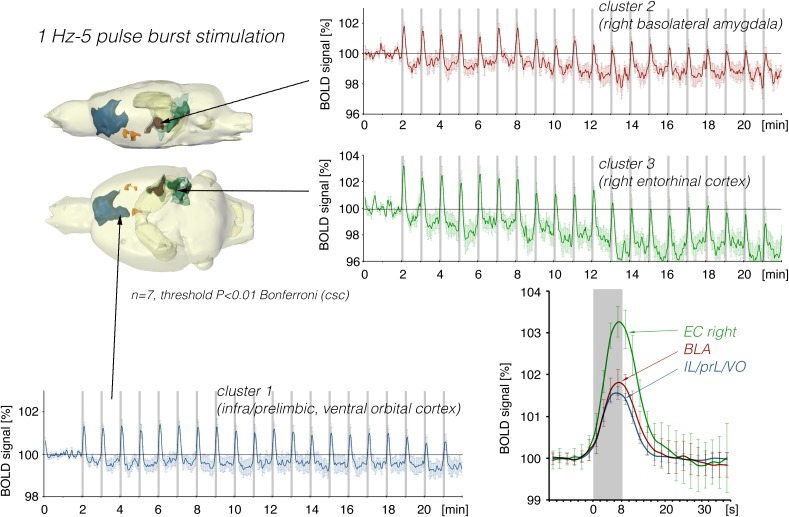
Summary of BOLD responses induced by stimulation of the right LEC with bursts of five high-frequency (100 Hz) pulses. Note that the spatial distribution of significantly activated voxels as well as the magnitude of BOLD responses is increased when compared to continuous 5 Hz pulse stimulation (Figure 2, 3). The magnitude of the BOLD responses in the amygdala and frontal cortex region were similar.

**FIGURE 5:**
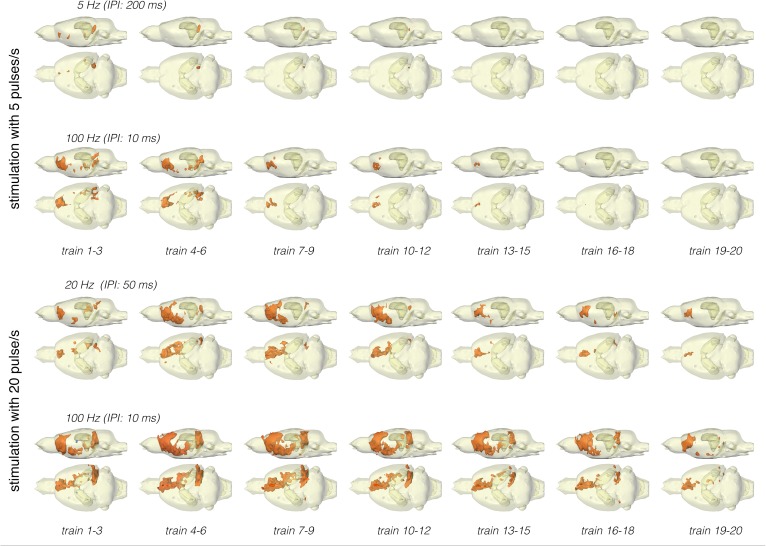
Development of significant BOLD responses during repetitive stimulation of the right LEC with different protocols. Independent of the stimulation protocol, the spatial distribution of significantly activated voxels varied during consecutive trains.

Stimulation of the LEC with the same number of pulses (but as short 100 Hz pulse bursts) resulted in a broader spatial distribution of significantly activated voxels when compared to continuous 5 Hz pulse stimulation (Figure 4). A second level analysis to map significant differences in BOLD activation between the two stimulation patterns revealed that an identical number of pulses given at high frequency bursts triggered significantly stronger activation in the infralimbic, prelimbic cortex, right piriform cortex (Pir), and nucleus accumbens (NAcc) (Figure 3). Under both conditions, individual BOLD responses declined during repetitive stimulation. During continuous 5 Hz stimulation, significant BOLD responses persisted with the longest duration in the entorhinal cortex region. During high-frequency pulse burst stimulation, BOLD responses in the IL/PrL/VO region persisted with the longest duration (Figure 5).

### Electrical Stimulation of the Lateral Entorhinal Cortex With 20 Pulses per Second

**FIGURE 6:**
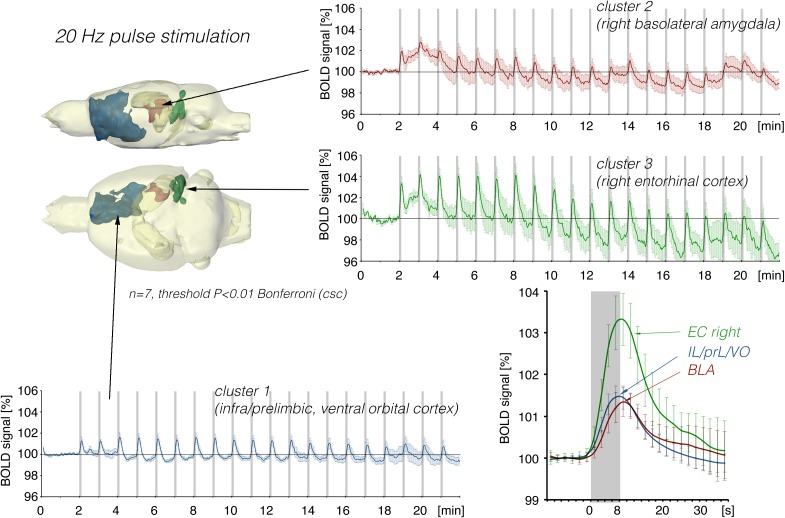
Summary of BOLD responses induced by stimulation of the right LEC with repetitive 20 Hz pulse trains. Under this condition, the spatial distribution of significantly activated voxels in the frontal cortex was increased compared to continuous 5 Hz stimulation (see Figure 2).

In a second set of experiments, the LEC was stimulated with a four-fold higher number of pulses per second. This increase in pulses not only increased the spatial distribution of significantly activated voxels but also the amplitude of the BOLD response (Figure 2, 6). In addition, under this stimulation condition, significant BOLD responses were also observed in the mPFC. Stimulation with continuous 20 Hz pulse trains occasionally caused neuronal after-discharges in the dentate gyrus. Accordingly, BOLD signals remained elevated after stimulation ceased and the averaged BOLD response appeared broadened. Presenting these 20 pulses again as a 100 Hz pulse burst further enlarged the spatial distribution of significantly activated voxels as well as the magnitude of BOLD responses (Figure 3, 6–10 and Table 1). Under this stimulation condition, significant BOLD responses were also detected in the contralateral entorhinal cortex.

**FIGURE 7:**
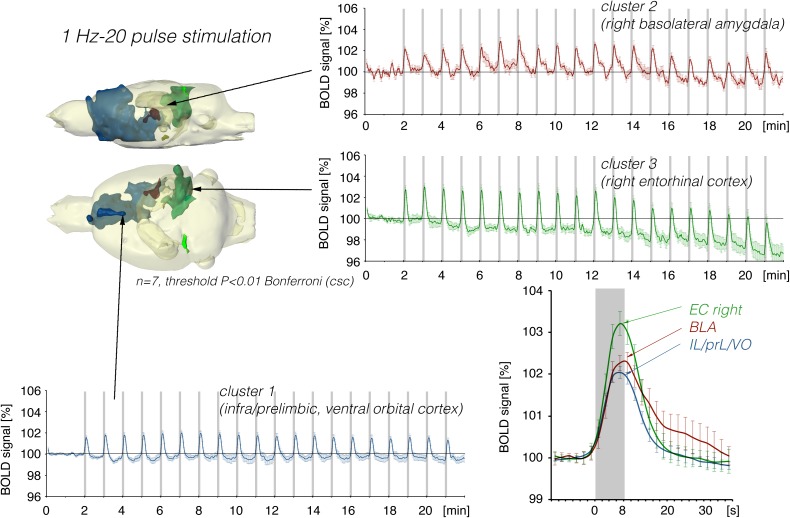
Summary of BOLD responses induced by stimulation of the right LEC with bursts of 20 high-frequency (100 Hz) pulses. Although the same number of pulses were applied as in Figure 5, the spatial distribution of significantly activated voxels increased as well as the magnitude in activation clusters 1 and 2.

**FIGURE 8:**
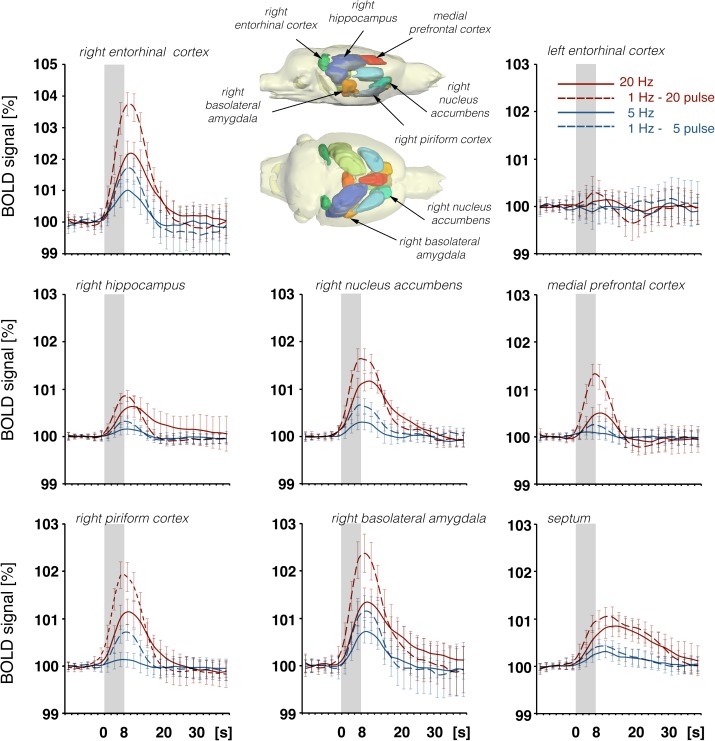
Summary of BOLD responses in individual VOIs during stimulation of the LEC with different stimulation protocols. VOIs of interest are depicted in the 3D rat brain template. Event-related BOLD responses represent average BOLD signal changes of all voxels in the appropriate VOI. Whereas BOLD responses in the septum and hippocampus mainly depended on the number of pulses, BOLD responses in the entorhinal cortex, piriform cortex, and basolateral amygdala mainly depended on the pulse frequency.

**FIGURE 9:**
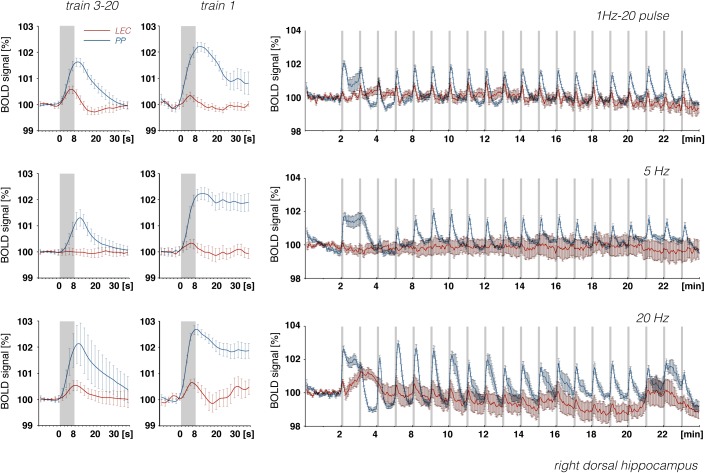
Development of BOLD signal changes in the right hippocampus during repetitive stimulation of the right LEC (red graphs) or the right perforant pathway (blue graphs) with different stimulation protocols. Note that the strongest BOLD responses were always induced by perforant pathway stimulation.

**FIGURE 10:**
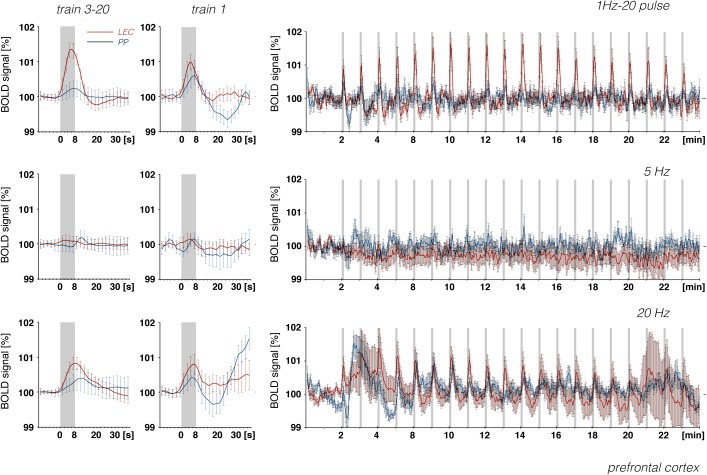
Development of BOLD signal changes in the prefrontal cortex during repetitive stimulation of the right LEC (red graphs) or the right perforant pathway (blue graphs) with different stimulation protocols. Note that the strongest BOLD responses were always induced by LEC stimulation.

**Table 1:** Summary of average BOLD responses in individual regions during electrical stimulation of the right LEC.

Region	20 Hz	1 Hz 20 pulse	5 Hz	1 Hz 5 pulse
HC right	100.63 ± 0.23	100.85 ± 0.12	100.16 ± 0.11	100.31 ± 0.11
HC right dorsal	100.56 ± 0.11	100.55 ± 0.15	100.01 ± 0.14	100.16 ± 0.11
HC left	100.08 ± 0.13	99.99 ± 0.09	100.00 ± 0.08	99.97 ± 0.12
HC left dorsal	100.00 ± 0.15	99.98 ± 0.13	100.03 ± 0.13	100.00 ± 0.13
EC right	102.15 ± 0.39	103.73 ± 0.36	101.01 ± 0.35	101.71 ± 0.44
EC left	100.14 ± 0.21	100.29 ± 0.34	100.00 ± 0.24	100.02 ± 0.38
striatum right	100.35 ± 0.14	100.41 ± 0.16	100.00 ± 0.10	100.11 ± 0.10
striatum left	100.02 ± 0.09	100.05 ± 0.12	100.02 ± 0.12	100.02 ± 0.13
NAcc right	101.16 ± 0.16	101.62 ± 0.23	100.30 ± 0.16	100.63 ± 0.16
NAcc left	100.19 ± 0.18	100.47 ± 0.11	100.04 ± 0.11	100.12 ± 0.15
septum	100.84 ± 0.22	101.05 ± 0.19	100.31 ± 0.14	100.42 ± 0.12
mPFC	100.50 ± 0.18	101.31 ± 0.23	100.10 ± 0.15	100.25 ± 0.18
VDB	101.16 ± 0.25	101.63 ± 0.18	100.38 ± 0.18	100.77 ± 0.21
VTA	100.28 ± 0.18	100.45 ± 0.15	100.13 ± 0.13	100.13 ± 0.16
right BLA	101.32 ± 0.31	102.37 ± 0.41	100.65 ± 0.29	101.14 ± 0.33
piriform cortex	101.15 ± 0.27	101.92 ± 0.28	100.14 ± 0.15	100.71 ± 0.17

*Significant BOLD signal changes are indicated in red.*

Stimulation of the perforant pathway with 20 pulses per second elicited significant BOLD responses in the dorsal right and left hippocampus and the right and left entorhinal cortex. In contrast to LEC stimulation, bursts of high-frequency pulse stimulation of the perforant pathway caused no more wide-spread BOLD responses when compared to continuous 20 Hz pulse stimulation (Figure 3).

## Discussion

Based on detailed anatomical studies in rats, it has been convincingly shown that lateral entorhinal neurons project to a variety of cortical and subcortical regions (Insausti et al., 1997; Agster et al., 2016). The current fMRI study confirms these extensive projections and extends these findings with the observation that, dependent on the frequency of entorhinal cortex stimulation, these anatomical connections become differently effective. In particular, activation of the LEC in the low theta frequency range (i.e., 5 Hz), beta frequency range (i.e., 20 Hz), and high gamma frequency range (i.e., 100 Hz) resulted in specific spatial distributions of significant BOLD responses. In general, increasing the stimulation frequency as well as the number of pulses resulted in the enlargement of significant BOLD responses. Thus, bursts of high-frequency pulses more efficiently generated BOLD responses in various target regions of the entorhinal cortex compared to the same number of pulses applied with a lower frequency.

Low-frequency pulse (i.e., 5 Hz) LEC stimulation elicited significant BOLD responses on the IL and BLA, whereas higher pulse frequencies were required to induce significant BOLD responses in the right hippocampus, dorsal mPFC (PrL, ACC), and piriform cortex. The apparent insensitivity of the dentate gyrus/hippocampus proper region was surprising, as these parts of the hippocampal formation are considered major targets of LEC projection neurons. One reason might be that electrical stimulation of the LEC only activates a small subset of neurons, which in turn only activates a small subset of neurons in the dentate gyrus and hippocampus proper. This is supported by the simultaneously recorded small electrophysiological responses in the dentate gyrus (Figure 1) that were in the range of previous studies (Roysommuti et al., 2003; Yaniv et al., 2003). These responses were clearly lower than responses that were induced by electrical stimulation of the perforant pathway, an axon bundle of entorhinal cortex neurons projecting to the dentate gyrus/hippocampus proper.

This might be due to the fact that the current required to directly activate neurons is proportional to the square of the distance between neuron and electrode tip (i.e., *I* = *K*r^2^; *I* is the current, *K* is a excitability constant, and r is the radius) (Tehovnik et al., 2006). As the excitability constant *K* depends on tissue, in particular on the axon size and myelination (summarized in Tehovnik et al., 2006), current injection in the perforant pathway activates more neurons than current injection in the gray matter of the entorhinal cortex.

In accordance with these electrophysiological differences, BOLD responses in the right dorsal hippocampus were also stronger during stimulation of the right perforant pathway when compared to the same stimulation of the right LEC (Figure 9). The fact that electrical LEC stimulation only affects a subset of neurons in the vicinity of the stimulation electrode also applies for the interpretation of BOLD signals in the amygdala complex as well as medial and dorsal frontal cortex regions, regions in which clear BOLD responses were induced. Therefore, the different BOLD responses in major target regions of the LEC could be mediated by: (1) differences in the threshold for spiking of neurons projecting to these regions, (2) differences in the processing of incoming pulses, and/or (3) regional differences in the efficacy of neurovascular coupling mechanisms. Without concurrent electrophysiological recordings in the amygdala complex and frontal cortex regions, it remains speculative as to why the magnitude of BOLD response varies in these major target regions of the LEC. In all experiments, the same parameters were applied to stimulate the LEC, i.e., pulses with 500 μA and 0.2 ms width; thus, the region of activated LEC neurons should be similar between the individual experiments. Nevertheless, the ratio of BOLD responses between different stimulation conditions was variable in individual regions. In the septal and hippocampal regions, the magnitude of BOLD response is roughly related to the number of pulses, whereas in the piriform cortex and amygdala complex, these responses are related to the number and frequency (Figure 7). This indicates that these regional different BOLD responses are mediated by distinct local signal processing. It also indicates that the formation of significant BOLD responses in the rat hippocampus requires the widespread synchronized activity of LEC neurons, whereas activation of only a subset of LEC neurons is sufficient to elicit significant BOLD responses in the amygdala complex and frontal cortex area.

Whereas electrical stimulation of a subset of LEC neurons triggered clear BOLD responses in the frontal cortex, similar stimulation of the perforant pathway only caused minor or even no BOLD responses in the frontal cortex region, although much stronger BOLD responses were elicited in the hippocampus (Figure 10). It appears that, in contrast to LEC projections, projections from the hippocampus proper/subiculum neurons, which are directly activated by perforant pathway fibers, are less efficient at triggering BOLD responses in the frontal cortex. This also supports recent findings that high-frequency pulse stimulation of Schaffer collaterals causes the formation of negative BOLD responses only in the mPFC (Bovet-Carmona et al., 2018) and that CA3 stimulation alleviates otherwise induced positive BOLD responses in the prefrontal cortex (Scherf and Angenstein, 2017). In particular, it appears that afferents from the LEC and CA1/subiculum elicit different BOLD responses in regions of the frontal cortex. This supports the assumption that the magnitude of a BOLD response depends on the quality of local signal processing rather than only the input activity.

In the context of using the entorhinal cortex as a target for deep brain stimulation to improve cognitive skills in the early stages of Alzheimer’s dementia (Stone et al., 2011; Hardenacke et al., 2013; Suthana and Fried, 2014; Bick and Eskandar, 2016; Xia et al., 2017), researchers should consider that variations in the applied pulse patterns might result in fundamentally different outcomes. However, it should also be noted that the current study used young healthy rats, and applying similar stimulation patterns in aged rats or in rat models for Alzheimer’s disease might cause other BOLD response patterns. Nevertheless, the present results indicate that the observed improvements in cognitive skills during or after deep brain stimulation of the EC might also crucially depend on concurrent activation of and/or interference with signal processing in the ventral/dorsal prefrontal cortex, as the activity in these prefrontal regions is mainly affected by lateral EC stimulation.

## Ethics Statement

The experiments were approved by the animal care committee of the State of Saxony-Anhalt (No.: 42502-2-1218 DZNE) and were performed according to the ARRIVE (Animal Research: Reporting *in vivo* Experiments) guidelines.

## Author Contributions

KK and LM performed the experiments. FA performed the experiments, analyzed the data, and wrote the manuscript.

## Conflict of Interest Statement

The authors declare that the research was conducted in the absence of any commercial or financial relationships that could be construed as a potential conflict of interest.
